# Supplementary material for: Neurogranin Regulates Adult-Born Olfactory Granule Cell Spine Density and Odor-Reward Associative Memory in Mice
Source: Int J Mol Sci. 2021 Apr 20;22(8):4269. doi: 10.3390/ijms22084269 (PMC8074334; doi:10.3390/ijms22084269)
Supplement: Supplementary file 1 [file ijms-22-04269-s001.zip › ijms-1169442-supplementary.pdf]

Supplementary materials

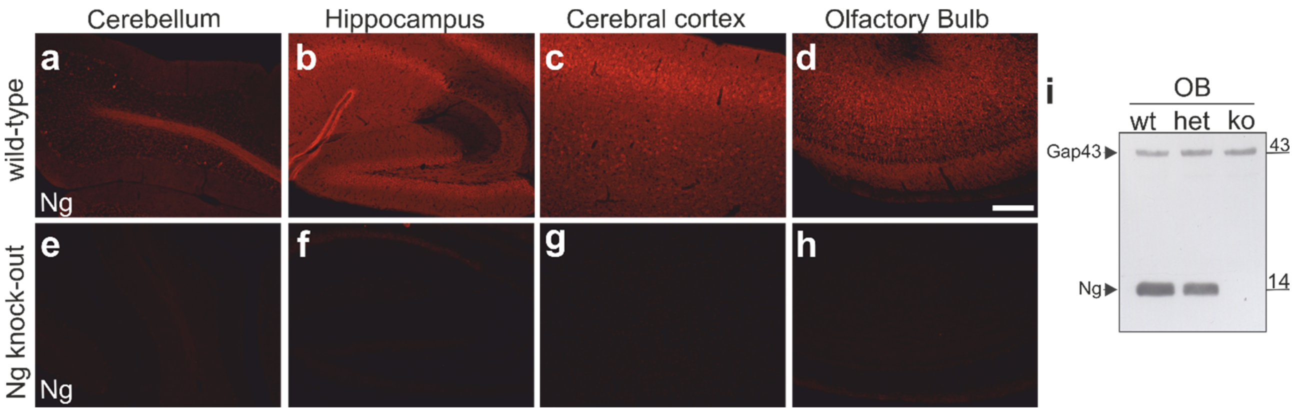

Supplementary Figure S1. Characterization of Ng expression in Ng-ko mice. (**a**–**h**) Characterization of Ng protein presence by immunofluorescence staining in different brain regions of wt (**a**–**d**) and Ng-ko (**e**–**h**) mice. Scale bar in d = 200 µm and applies to (**a**–**h**). (**i**) Western blot analysis on OB extracts indicates the absence of a Ng-positive band (14 KDa) in Ng-ko mice. A band at 43 KDa corresponding to Gap43 is present in all three genotypes at comparable intensity.

	GL	EPL	GCL	SVZ	TOT
wt	1,813 ± 0,025	1,979 ± 0,068	2,341 ± 0,036	0,067 ± 0,024	6,200 ± 0,105
het	1,848 ± 0,029	2,194 ± 0,091	2,608 ± 0,053	0,055 ± 0,005	6,705 ± 0,168
Ng-ko	1,822 ± 0,001	2,039 ± 0,178	2,428 ± 0,004	0,064 ± 0,004	6,353 ± 0,183

Supplementary Table S1. Volume of OB layers. The volume (mm^3 ± standard deviation) of each OB layer is shown in wild type (wt), heterozygotes (het) and Ng-knock-out mice (Ng-ko); n = 2 for each genotype.
